# Inter-Cellular Variation in DNA Content of *Entamoeba histolytica* Originates from Temporal and Spatial Uncoupling of Cytokinesis from the Nuclear Cycle

**DOI:** 10.1371/journal.pntd.0000409

**Published:** 2009-04-07

**Authors:** Chandrama Mukherjee, Shubhra Majumder, Anuradha Lohia

**Affiliations:** Department of Biochemistry, Bose Institute, Kolkata, India; New York University School of Medicine, United States of America

## Abstract

Accumulation of multiple copies of the genome in a single nucleus and several nuclei in a single cell has previously been noted in *Entamoeba histolytica*, contributing to the genetic heterogeneity of this unicellular eukaryote. In this study, we demonstrate that this genetic heterogeneity is an inherent feature of the cell cycle of this organism. Chromosome segregation occurs on a variety of novel microtubular assemblies including multi-polar spindles. Cytokinesis in *E. histolytica* is completed by the mechanical severing of a thin cytoplasmic bridge, either independently or with the help of neighboring cells. Importantly, cytokinesis is uncoupled from the nuclear division cycle, both temporally and spatially, leading to the formation of unequal daughter cells. Sorting of euploid and polyploid cells showed that each of these sub-populations acquired heterogeneous DNA content upon further growth. Our study conclusively demonstrates that genetic heterogeneity originates from the unique mode of cell division events in this protist.

## Author Summary

Proliferating eukaryotic cells regulate their DNA synthesis, chromosome segregation, and cell division with great precision so that daughter cells are genetically identical. Our study demonstrates that in proliferating cells of the protist pathogen *Entamoeba histolytica* re-duplication of DNA followed by segregation on atypical and diverse microtubular structures is frequently observed. In this parasite, cell division is erratic, so that each daughter cell may contain one or more nuclei and sometimes no nuclei. This uncoupling of cell cycle events and survival of daughter cells with unequal DNA contents leads to genetic heterogeneity in *E. histolytica*. Our study highlights the inherent plasticity of the *Entamoeba* genome and the ability of this protist to survive in the absence of strict regulatory mechanisms that are a hallmark of the eukaryotic cell cycle.

## Introduction

Eukaryotic cells undergo two major events during proliferation- the nuclear cycle, when the whole genome is duplicated and segregated equally into two nuclei and cytokinesis, the physical separation of a mother cell into two daughter cells. Sequential progression of events during the cell cycle is enforced by checkpoint proteins [1] that also ensure the spatial and temporal coordination of mitosis with cytokinesis [2]–[4]. While this paradigm is true for most organisms that have been studied, it is becoming increasingly clear that diverse groups of eukaryotes, including plants, ciliates, *Drosophila*, and protists, show striking differences in regulating the transmission of genetic information during proliferation [5]. Many of these organisms tolerate large variations in DNA content during different life cycle stages such that typical cell cycle checkpoints are altered. *Entamoeba histolytica* is one such protist parasite that proliferates in the intestinal lumen of human beings often causing severe disease in the host. Indeed this parasite is responsible for millions of cases of dysentery and liver abscess world wide especially in developing countries [6].

Axenically grown *E. histolytica* cells exhibit variations in DNA content, which may vary several folds within a population [7]. Multiple genome complements may be present in a single nucleus or distributed over multiple nuclei in a single cell [8],[9]. Re-duplication of the genome and de-linking of S-phase from cytokinesis were identified as two of the reasons for the generation of polyploidy in these trophozoites [8]. A significant number of *E. histolytica* trophozoites with multiple nuclei were observed in infected intestinal tissue suggesting this is an intrinsic property of this parasite and not due to *in vitro* culture conditions [10]. Remarkably, under different growth conditions such as switching from xenic to axenic growth leads to a significant increase in the nuclear DNA content of *E. histolytica* trophozoites [10]. Similar variation in nuclear DNA content was also observed in the related reptilian parasite *E. invadens* during conversion of cysts to trophozoites and vice versa [10]. These observations clearly indicate the inherent plasticity of the *Entamoeba* genome [10] and the ability of this protist to survive in the absence of strict regulatory mechanisms that are a hallmark of the eukaryotic cell cycle.

Most eukaryotes segregate their genomes once duplication is complete. While more than 2n genome content accumulates in some *E. histolytica* cells due to multiple rounds of DNA duplication, it has not been clear how polyploid amoebae partition their genomes. Bipolar microtubular spindles are not frequently visible in *E. histolytica* [11]. Rather, several electron microscopic studies have reported atypical organization of microtubules (MTs) during nuclear division [12]–[14]. Thus, the possible cell-cycle mechanism underlying the dynamic variation in DNA content of *E. histolytica* cells during their axenic growth include- a) multiple cycles of replication of the genome without nuclear or cell division, b) nuclear division without cytokinesis and c) polyploidization from over-replication of the genome followed by segregation on multipolar mitotic spindles.

In this study, we demonstrate: a) the formation of atypical microtubular structures during genome segregation; b) multiple microtubule organizing centers (MTOCs) and multi-polar spindles are formed in polyploid nuclei; c) cell division is spatially and temporally uncoupled from the nuclear cycle; d) cell division can be asymmetric, thereby producing either uni-nucleate, multi-nucleate or anucleate daughter cells. In summary, asynchrony between nuclear division and cytokinesis in a fraction of the population combined with segregation of the polyploid genome on multi-polar spindles accounts for the extreme variation of genomic DNA content in individual *E. histolytica* cells in axenic culture.

## Materials and Methods

### Cell culture and maintenance

*E. histolytica* HM-1:IMSS trophozoites were maintained axenically and routinely sub-cultured every 48–72 h in TYI-S-33 medium containing 10% adult bovine serum at 37°C [15].

### Cell synchronization by serum starvation

*E. histolytica* HM-1:IMSS cells were sub-cultured every 24 h for 3–4 days followed by serum starvation [8]. Adult bovine serum was added (10%) after 12–13 h of serum starvation. Cells were then withdrawn at different times as indicated and fixed.

### Scanning cytometry to determine nuclear DNA content

Ethanol fixed cells were stained with 4′-6-Diamidino-2-phenylindole (DAPI, 0.1 µg/ml, Sigma) for 10 min, washed once with 1× PBS and then scanned for DNA content of individual nuclei [10] under a 40× oil objective (numerical aperture 1.3) of a Zeiss Axiovert 200 M fluorescence microscope fitted with the MetaCyte scanning cytometer (Zeiss, Germany). A minimum of 2000 nuclei was scanned for each sample and analyzed by Metafer4 software (Zeiss, Germany). The DAPI fluorescence values (x-axis) were represented as histograms. The scan yields varying numbers of nuclei with different fluorescence values. The number of nuclei on the y-axis was represented as a fraction of the highest number of nuclei obtained in any one sub-class of each scan.

### Immunofluorescence and confocal microscopy

*E. histolytica* HM-1:IMSS cells were grown on coverslips in 24-well plates at 37°C, fixed directly with warm 3.7% formaldehyde for 15 min and permeabilized with 0.1% Triton X-100 for 10 min. Fixed cells were stained with polyclonal anti-Eh β-tubulin antibody [11] followed by tetramethyl rhodamine isothiocyanate (TRITC) conjugated anti-rabbit secondary antibody (1∶200; Jackson Laboratories, USA). For visualization of actin filaments, cells were incubated with Alexa Fluor 488 conjugated phalloidin for 30 min (Molecular Probes, Invitrogen, USA). Images were acquired with (i) 63× Plan-Apochromat 1.4 oil differential interference contrast (DIC) objective (numerical aperture 1.4) in a Zeiss LSM 510 Meta confocal microscope equipped with a 488 nm argon laser and a 543-nm He/Ne laser and were analyzed with the LSM Meta 510 software package (Zeiss, Germany) or (ii) a 40× oil objective (numerical aperture 1.3) in an Axiovert 200 M fluorescence microscope using Z-stacking and analyzed by deconvolution (Axiovision v4.6). DNA was stained with DAPI (0.2 µg/ml, Sigma) for 30 min.

### Real-time microscopy

*E. histolytica* HM-1:IMSS trophozoites were plated on 35-mm plastic culture dishes filled with growth medium at 37°C. After the cells adhered, the medium was replaced with fresh growth medium. The dish was kept inside an incubator (Tempcontrol 37-2 *digital*, Zeiss, Germany) at 37°C and under 5% CO_2_ flow system (PeCon GmbH, Erbach, Germany) which was fitted to the Axiovert 200 M fluorescence microscope (Zeiss, Germany). Cells were visualized under a 20× phase contrast objective. The time-lapse images were captured with 1 sec interval for the indicated time, then analyzed and further processed by Axiovision v4.6 software (Zeiss, Germany).

### Cell sorting

Asynchronously growing *E. histolytica* HM-1:IMSS cells (1×10^8^ cells) were harvested 48 h after sub-culture, washed with 1× PBS and incubated with 1 µM DNA binding dye- Vybrant DyeCycle Orange for 1 h (Molecular Probes, Invitrogen, USA). Cells were passed through a 40 µm nylon mesh (Becton Dickinson, USA) to remove aggregates and debris before sorting the cells. Vybrant DyeCycle Orange stained cells were excited at 488 nm in a flow cytometer (FACSAria, Becton Dickinson, USA) and emission was measured through a 585/42 Band Pass filter for analyzing the DNA content. On the basis of DNA content analysis (FACSDIVA 6.0 software, Becton Dickinson, USA), cells were demarcated in four different electronic gates and sorted at 4°C. The sorted cells were washed with 1× PBS and resuspended in 2 ml TYI-S-33 medium. Half the cells were inoculated into growth medium and harvested after 3 days. The remaining cells were fixed in 70% ethanol for analysis of the nuclear DNA content and number of nuclei in each cell.

## Results

### Endo-replication and uncoupling of the nuclear division cycle from cell division are both seen in synchronized cells

**Figure 1:**
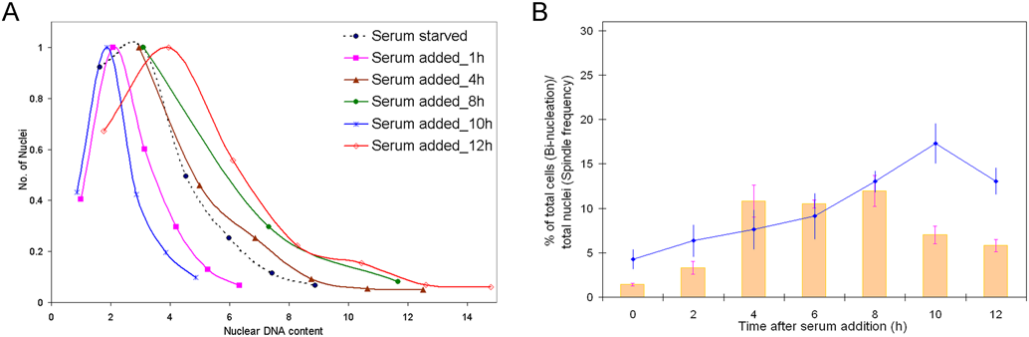
S-phase and chromosome segregation are coupled to nuclear division but the nuclear cycle is uncoupled from cell division. (A) *E. histolytica* HM-1:IMSS cells were fixed after 12–13 h serum starvation and after serum re-addition (1 h–12 h). The nuclear DNA content was deduced from DAPI fluorescence intensities and shown as individual histograms (X-axis). Y-axis represents the number of nuclei as a fraction of the highest number of nuclei obtained in any sub-class of each scan. Representative data from three independent experiments are shown. The nuclear DNA content profiles between 2 h to 4 h and 5 h to 9 h were similar and therefore only 4 h and 8 h have been shown. (B) Number of bi-nucleated cells (line) and the frequency of distinct microtubular assemblies (bar) were scored after serum starvation (0 h) and serum re-addition (2 h–12 h). At least 150–200 cells were analysed at each time point and an average of three independent experiments are shown with error bars indicating±S.D.

DNA synthesis of *E. histolytica* is arrested when cells are incubated in serum free media for at least 12 h, followed by re-initiation within 2 h after addition of serum [7],[8]. Endo-replication of the genome in the population can be seen following this synchronization protocol [8]. We have now examined the fates of individual nuclei following this synchronization. Progression of S-phase was monitored by estimating the nuclear DNA content in DAPI stained cells. Nuclear division was estimated from the increase in bi-nucleated cells while cell division was estimated from counting cell numbers. Our data show that after 12 h of serum starvation, *E. histolytica* nuclei contain heterogeneous amounts of DNA. 1 h after addition of serum the nuclei show a dramatic homogenization and reduction of DNA content (Figure 1A). This early homogenization likely results from nuclear division in polyploid nuclei. An earlier report identified two copies of selected loci using fluorescent in situ hybridization and estimated amoeba to be diploid [16] while other studies have demonstrated that the DNA content can vary several fold in proliferating amoebae so that single cells may contain up to 10n or 12n [7],[10]. We assigned 1n genome content to the lowest nuclear DNA content after homogenization. Compared to nuclei 1 h after the addition of serum (1n–2n), the average DNA content of *E. histolytica* nuclei increased 2 fold in 80–90% of the nuclei between 2 h and 8 h after addition of serum (Figure 1A). Between 8 h and 10 h after addition of serum, the nuclear DNA content again showed reduction and homogenization to a level similar to that observed at 1 h after serum addition. This suggests completion of chromosome segregation and nuclear division within 10 h. The homogenous euploid nuclear DNA content at 1 h and 10 h after serum addition marks the temporal boundaries of a single nuclear cycle. The nuclear DNA content increased again after 10 h suggesting that another cycle had been initiated. In each of these cycles, 10–20% of the nuclei accumulated greater than 4n DNA content (Figure 1A) suggesting that some nuclei undergo endo-replication without nuclear division. This polyploid population was absent at 1 h and 10 h after serum addition, suggesting that either chromosome segregation occurs more than once in a single nuclear cycle in the polyploid nuclei or the polyploid nuclei can segregate multiple copies of the genome simultaneously.

In order to assess whether chromosome segregation was coupled with nuclear division, we scored the number of microtubular assemblies and bi-nucleated cells in conjunction with changes in DNA content. Earlier studies have demonstrated that microtubules were mostly nuclear [11],[12],[17]. After 12 h of serum starvation, tubulin was dispersed in the nucleus without any obvious structure in most cells. Microtubular assemblies or structures began to appear 2 h after serum addition, continued to increase in number up to 8 h, and then decreased markedly at 10 h (Figure 1B). Bi-nucleated cells were highest (∼20%) at 10 h after serum addition in these cells (Figure 1B). Thus for 20% of the cells nuclear DNA replication was immediately followed by segregation of the chromosomes into daughter nuclei.

Importantly, cell numbers increased gradually rather than in a step-wise fashion coinciding with increase in nuclear number (Figure S1A). While cell density affected the rate of increase in cell number (Figure S1B), the temporal progression of the nuclear division cycle was independent of these factors. This suggests that although nuclear division is coupled to DNA synthesis and chromosome segregation, cell division is random and not obligately linked to nuclear division in these synchronized *E. histolytica* cells.

### Genome segregation may occur on monopolar, bipolar or multi-polar microtubular spindles in *E. histolytica*

**Figure 2:**
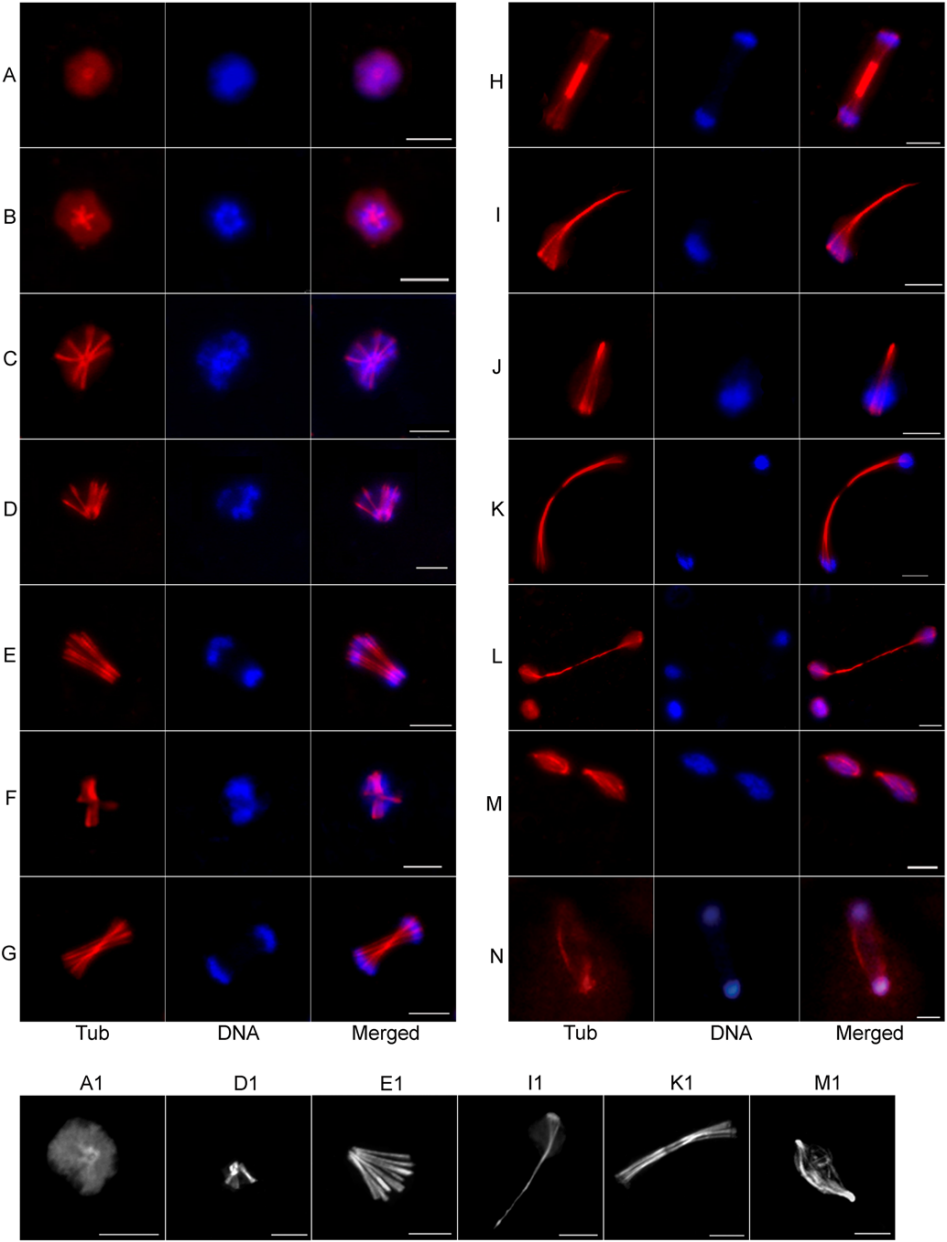
Diverse microtubular assemblies in *E. histolytica*. Synchronized *E. histolytica* HM-1:IMSS cells were stained with anti-Eh β-tubulin antibody and visualized under Axiovert 200 M flourescence microscope. Representative images of a radial assembly (A), variety of monopolar (B–L) and bipolar (M–N) microtubular assemblies are shown. Chromosomal DNA was stained with DAPI. In panel M, two bipolar MT spindle intermediate are seen in a single cell. Cells from this experiment were visualized under a laser scanning confocal microscope for finer details of the microtubular structures. Comparable confocal images of the MT assemblies shown in panel A, D, E, I, K and M are shown as A1, D1, E1, I1, K1 and M1 respectively. Bar in each panel represents 5 µm.

During the course of a mitotic cycle, we observed different MT assemblies and structures in the amoeba nucleus. Commonly, anti-Eh β-tubulin antibody showed a diffuse nuclear stain and MT structures were not visible in most nuclei. In some nuclei, a single pole-like body was observed at the center (Figure 2A). Confocal microscopic analyses clearly showed a central ring-like arrangement of MTs with radially disposed short MT fibers (Figure 2A1). It has been shown that Eh γ-tubulin formed a similar ring-like arrangement at the center of *E. histolytica* nuclei thus defining the pole-like body as ‘MTOC’ in these cells [17]. During progression of the nuclear cycle we identified a variety of MT spindle-like assemblies that likely originated from the MTOC and radial MTs described above. The arrangement of chromosomes on these MT structures suggested that these structures were intermediates formed during genome segregation. Figure 2B–E (corresponding confocal images Figure 2D1 and 2E1) show MT fibres emanating from a single pole that may be an extension of the central pole seen in Figure 2A. Chromosomal DNA appears to have segregated at an early stage after attachment to the central pole and then been subsequently pushed apart at one end of the uni-directionally extending MT fibers (Figure 2C–E). These structures were observed in 25–30% of the spindle intermediates.

We also observed several non-conventional MT structures - a) with DNA at the two ends of bi-directionally extending MT fibers (Figure 2F–H) and b) with DNA bound at one end of several radiating MT fibers that bundled together at the other end (Figure 2I–J and 2I1). Segregation appears to be completed on extended MT structures where the elongated MTs have disappeared from the centre (Figure 2K–L and 2K1). Bipolar spindle like structures were also observed but at a low frequency (3–5% of the MT structures) compared to the monopolar intermediates. Chromosomal DNA was distributed longitudinally over the bipolar spindle or at the two poles (Figure 2M–N and 2M1).

The most frequent structures visible at 2 h–4 h after serum addition were the intermediates shown in Figure 2I–J (50–55% of the MT structures). Based on the different MT structures observed during progression of the nuclear cycle, it is likely that genome segregation occurs either by extension of a radial monopolar assembly (Figure 2C–E) or by lateral separation of MT fibers that are joined at a distal end (Figure 2I and 2J). It is unclear whether the MT structures shown in Figure 2F–H originated from monopolar assemblies or duplication of a MTOC. Instead of chromosomal segregation due to pole-ward contraction of MT fibers, the MT structures in amoeba nuclei suggest either a) separation of chromosomal DNA on the growing end of MTs, b) lateral separation of duplicated chromosomes and MTs, or c) longitudinal movement of chromosomes on elongated MTs.

**Figure 3:**
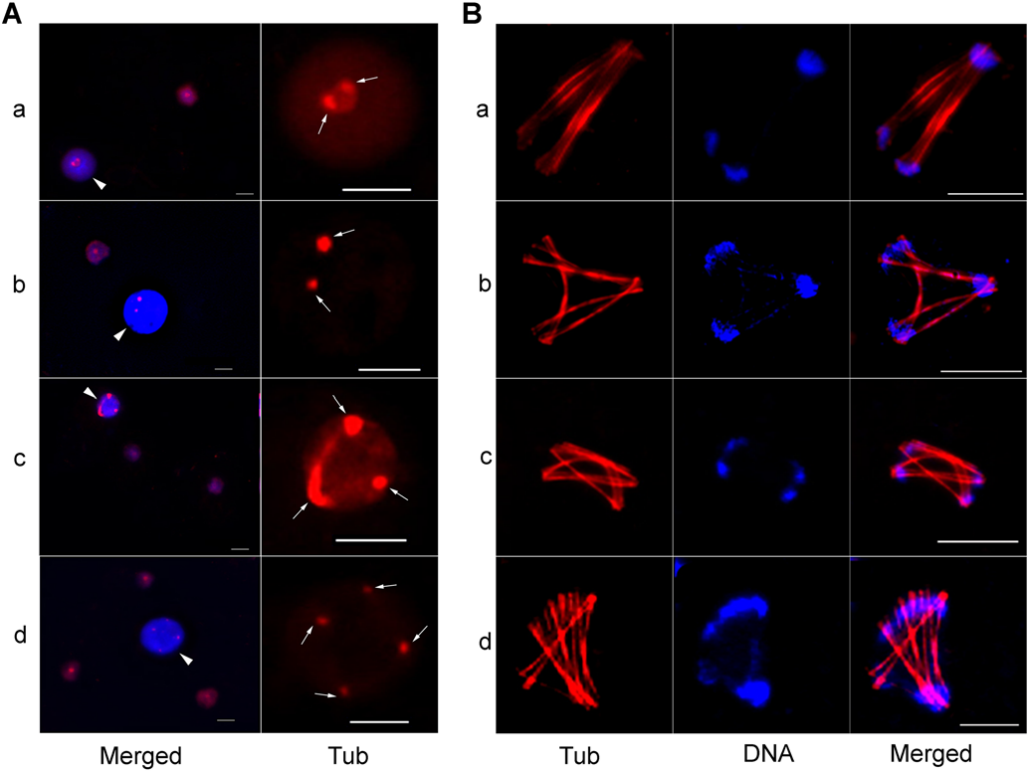
Multiple MTOCs in polyploid nuclei may lead to multi-polar spindles. (A) Anti-Eh β-tubulin antibody (red) and DAPI (blue) stained *E. histolytica* HM-1:IMSS cells showing several nuclei with high DNA content (arrowhead) compared to other nuclei (a–d, ‘Merged’ panel,). The presence of two (a, b), three (c) and four (d) MTOC or poles (arrows) in these polyploid nuclei are shown by the staining with anti-Eh β-tubulin antibody with higher magnification (‘Tub’ panel). (B) Representative images of tri-polar (a, b), tetra-polar (c) and multi-polar (d) MT assemblies are shown. Bar represents 5 µm.

Importantly, several nuclei with high DNA content were seen that contained two, three or four MTOCs (Figure 3A). Bipolar spindle intermediates likely originated from duplication of the central pole (Figure 3A-a, b). Interestingly, tri-polar, tetra-polar and multi-polar spindles were also seen in several cells (Figure 3B). It is possible that these multi-polar spindles originated from the multiple MTOCs in polyploid nuclei and were used to segregate multiple copies of the genome simultaneously. This could be one of the mechanisms for the segregation of multiple genome copies in polyploid nuclei during a single nuclear cycle.

In summary, *E. histolytica* uses novel MT structures for chromosome segregation mostly originating from a single pole. Multi-polar spindles are also observed and may account for the rapid generation of DNA content heterogeneity from polyploid cells. Another unique feature is the use of multiple modes of segregation, as suggested by the diverse MT structural intermediates and inter-cellular variation in DNA content.

### Cytokinesis occurs by scission of a cytoplasmic bridge and may require helper cells

No significant increase in the number of dividing cells at any time after completion of the nuclear cycle was observed in serum synchronized *E. histolytica* (data not shown). Therefore, based on earlier reports [18], we provided fresh growth medium to the culture to induce an increase in division events. Using real-time microscopy we identified at least two modes of cytokinesis. Cytoplasmic constriction was initiated at random sites generating two dividing parts of a cell that pulled away from each other, forming a thin cytoplasmic bridge that was eventually severed (Video S1 and Figure S2A). In many dividing cells, the complete scission of the cytoplasmic bridge utilizes the assistance of helper cells. In such cases, the helper cells, migrated through the connecting cytoplasmic bridge and ensured the mechanical rupture of the ‘connector’ (Video S2 and Figure S2B). From five independent experiments, we recorded 40 cell division events by real-time microscopy, after cytoplasmic constriction was initiated. Our results showed that one third (14 out of 40) of *E. histolytica* cell division events were assisted by helper cells while 45% (18 out of 40) involved independent mechanical severance of the cytoplasmic bridge. We also discovered that 20% (8 out of 40) of the cell division events in *E. histolytica* were not completed. In these cases, the connecting cytoplasmic bridges became unusually long and thin but were not finally severed. Instead, the cytoplasmic bridge contracted and the two ‘dividing halves’ fused together without division (Video S3).

Thus, besides the heterogeneity in chromosome segregation, and the lack of synchrony between nuclear division and cytokinesis, heterogeneity in modes of cytokinesis and frequent failure of completion of cytokinesis even after initiation, contribute to the genetic heterogeneity that arises in a population of *E. histolytica*.

### Heterogeneity in sites of cytokinesis adds to the genetic heterogeneity

**Figure 4:**
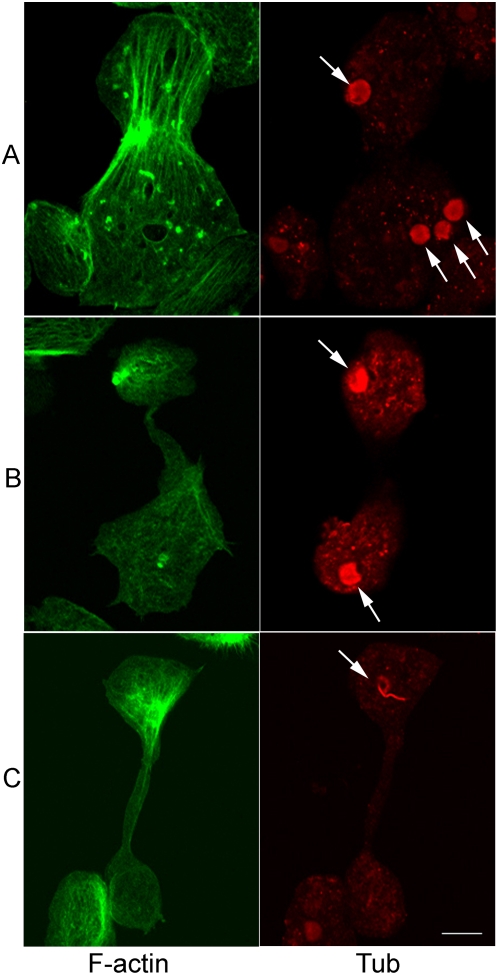
Cytokinesis is asymmetric and irregular in *E. histolytica*. *E. histolytica* trophozoites were grown on coverslips and stained for visualizing F-actin and nuclear MT assemblies (arrow). Representative images of different dividing cells are shown. Longitudinal F-actin fibers run through the constriction during initial stage of division (A) but are absent when the cytoplasmic bridge is extended (B, C). While dispersed Eh β-tubulin staining was observed in most of the nuclei during cell division (A, B), MT spindle intermediates could also be seen in some cells (C). The distribution of nuclei in the two dividing parts could be symmetric (B) or asymmetric (A). Cytokinesis may be initiated in a cell with one (C), two (B) or four (A) nuclei. Bar is 10 µm.

**Figure 5:**
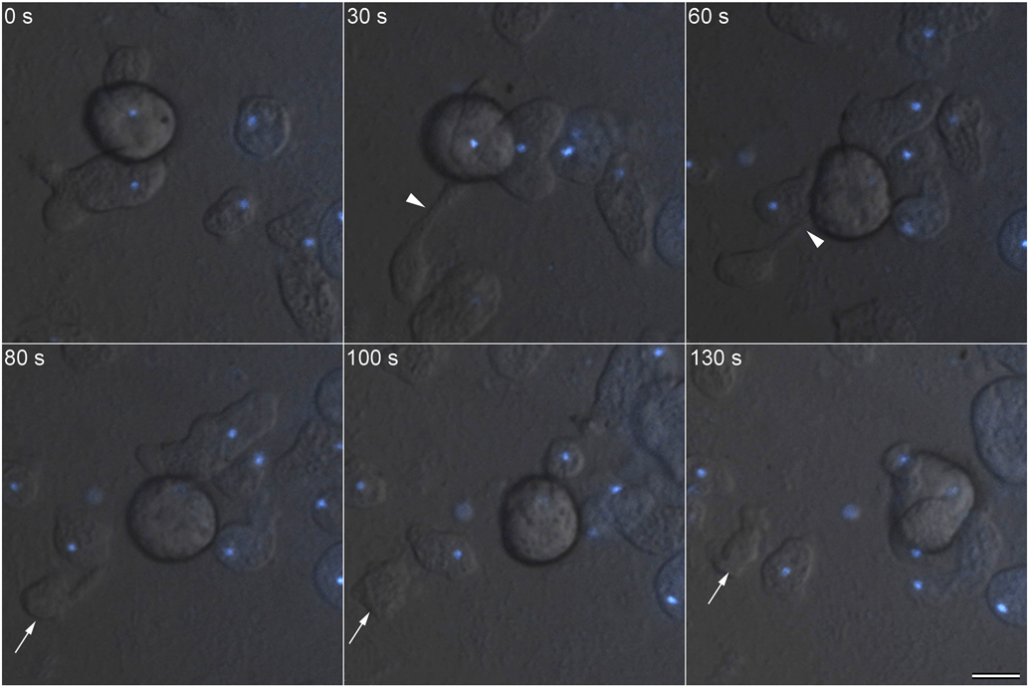
Asymmetric and erratic cell division leads to the formation of ‘anucleate’ daughter cells. Time-lapse images recorded during a cell division event in live *E. histolytica* labeled with the vital dye Hoechst 33342 are shown. Initiation of cytoplasmic bridge (arrowhead) in a uni-nucleated cell led to the formation of an anucleate daughter cell (arrow). Bar represents 20 µm.

In most organisms, the precise location of the cytokinesis apparatus between the two daughter nuclei is observed. We therefore, examined where the cytokinesis furrow was located in amoeba cells relative to locations of the daughter nuclei and how the reorganization of cytoplasmic actin assembly takes place in dividing cells. Longitudinal actin fibers perpendicular to the constriction site along with tiny F-actin patches were seen in cells likely to divide (Figure 4). Importantly, dividing cells may contain one or more nuclei in each ‘daughter’. Most of the events highlighted the randomness of cytokinesis site selection relative to the location of the daughter nuclei (Figure 4). In some cases, the nuclei may move away from their original position after initiation of cell division so that both nuclei are on the same side of the division site and thus ‘anucleate’ cells are generated. Anucleate cells may also form after erratic division of uni-nucleated cells (Figure 5). This inefficient and irregular cell division is likely responsible for the formation of heterogeneous daughter cells with variable numbers of nuclei in a population of *E. histolytica*. Thus, spatial uncoupling of cell division with nuclear division adds to the genetic heterogeneity in *E. histolytica*.

### Euploidy, polyploidy and variations in nuclear number are inherent features of the *E. histolytica* cell cycle

**Figure 6:**
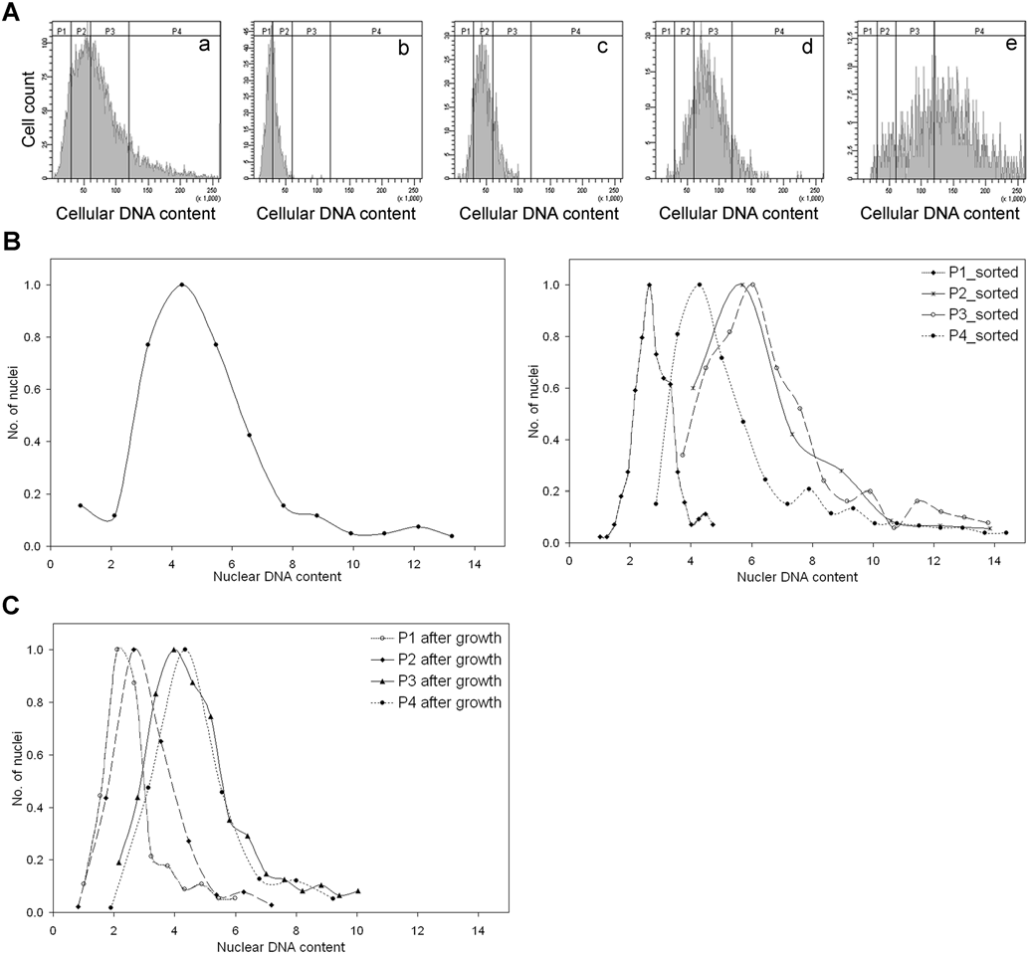
Euploidy, polyploidy and variations in nuclear number are inherent features of the *E. histolytica* cell cycle. (A) Cellular DNA content of 48 h grown live cells was analysed before sorting (a). Cells were sorted on the basis of increasing DNA content (P1–P4) and reanalyzed (b–e). (B) Ethanol fixed pre-sorted cells (left panel) and sorted sub-populations (P1–P4, right panel) were stained with DAPI to estimate the nuclear DNA content. The DAPI fluorescence intensities were normalized against the lowest value of P1 cells and are shown as individual histograms. Y-axis represents the number of nuclei as a fraction of the highest number of nuclei obtained in any sub-class of each scan. (C) Sorted sub-populations (P1–P4) of *E. histolytica* cells were grown in fresh TYI-S-33 medium. Fixed cells from the P1–P4 sub-populations were stained with DAPI and the nuclear DNA content measured. Fluorescence intensities for each set were normalized and shown as individual histograms. Y-axis represents the number of nuclei as a fraction of the highest number of nuclei obtained in any sub-class of each scan. Representative data from three independent experiments are shown.

In order to determine if the difference in DNA content was a stable property of both euploid and polyploid cells, we separated cells on the basis of their DNA content and followed the DNA content of the progeny. Log phase *E. histolytica* trophozoites were stained with the vital dye Vybrant orange and sub-populations of live amoebae with varying DNA contents were isolated using a fluorescence activated cell sorter. The fluorescence values representing cellular DNA content profile of unsorted cells showed a broad distribution (Figure 6A, a). Electronic gates (P1–P4) were set (Figure 6A, a) and cells were separated according to differences in DNA content. Figure 6A, b-e shows the cellular DNA content profile of the different sub-populations that were recovered after sorting. The sub-populations P1–P4 were analyzed for multi-nucleated cells and nuclear DNA content and compared with the unsorted cells. Table 1 shows that while the unsorted cells consisted of approximately 92% uni-nucleated cells and 8% bi- and multi-nucleated cells, sorted P1 and P2 cells were mostly uni-nucleated (92–96%) with only a small percentage of bi- and multi-nucleated cells (4–8%). On the other hand, P3 and P4 cells contained a significantly higher number of bi- and multi-nucleated cells (13–20%).

**Table 1:** Percentage of *E. histolytica* cells with one, two or greater than two nuclei in unsorted and sorted sub-populations of cells.

Sample	% of uni-nucleated cells±S.D.		% of bi-nucleated cells±S.D.		% of multi-nucleated cells±S.D.	
Unsorted	92.0±0.1		6.8±0.1		1.1±0.3	
	**After sorting**	**After growth**	**After sorting**	**After growth**	**After sorting**	**After growth**
P1	95.5±0.5	85.0±1.0	3.0±1.0	12.5±0.5	0.4±0.3	2.2±0.8
P2	91.5±1.5	91.0±1.0	7.3±1.3	7.65±0.6	0.85±0.1	0.4±0.3
P3	87.0±0.1	91.5±0.5	10.7±0.6	7.7±0.3	1.7±0.4	0.5±0.1
P4	82.0±2.0	89.5±0.5	17.0±0.4	8.6±0.4	2.7±0.4	1.5±0.5

The number of nuclei per cell was counted from the ethanol-fixed and DAPI stained cells. At least, 500 cells were counted at each time to determine the percentage of uni-nucleated, bi-nucleated and multi-nucleated (>2) cells. Average obtained from three independent experiments was shown with indicated standard deviation (S.D.).

A comparison of the nuclear DNA content in the unsorted cells showed a broad distribution of nuclear DNA content from 1n to 6n while about 15–20% nuclei contained greater than 6n DNA content (Figure 6B, left). P1 cells showed 1n–4n nuclear DNA content, P2 cells showed 4n–14n nuclear DNA content while P3 and P4 cells showed 3n–14n nuclear DNA content. (Figure 6B, right). To investigate whether the isolated sub-populations of P1–P4 cells would retain their DNA content and nuclear number phenotype after growth, the sorted cells were incubated in fresh medium at 37°C. Following an initial lag of 24–36 h (possibly due to the physiological stress induced by cell sorting), the cell numbers subsequently increased in all the tubes. After 72 h all the sorted sub-populations had undergone at least 2 doublings (final cell density ∼1–3×10^5^ cells per ml in each tube). It was observed that P1 cells had a higher percentage of bi-and multi-nucleated cells while P4 cells showed a homogenous population of uni-nucleated cells after growth (Table 1). Changes in the number of bi- and multi-nucleated cells were observed in P2 and P3 cells also. After growth the P2–P4 populations showed a reduction in the number of 10n–14n nuclei (Figure 6C). Therefore *E. histolytica* is programmed to continuously generate genetic heterogeneity in both nuclear number per cell and DNA content per nucleus, and this program is equally active in the polyploid and the euploid cells.

## Discussion

In this study, we have discovered that genetic heterogeneity in *E. histolytica* cells results from multiple modes of genome segregation during nuclear cycle, asynchrony between nuclear division and cytokinesis along with variation in nuclear DNA content.

### Different microtubular structures suggest variable and atypical modes of chromosome segregation in *E. histolytica*

Although recent studies have reported the infrequent occurrence of bipolar MT spindles [11],[19] in *E. histolytica* cells, metaphase-like equatorial alignment of condensed chromosomes or kinetochores could not be identified in amoeba [12],[14] while anaphase and telophase were identified on the basis of nuclear shape [13]. Indirect immuno-fluorescence showed nuclear microtubular assemblies with fibers radiating from a central region in most *E. histolytica* cells [17].

**Figure 7:**
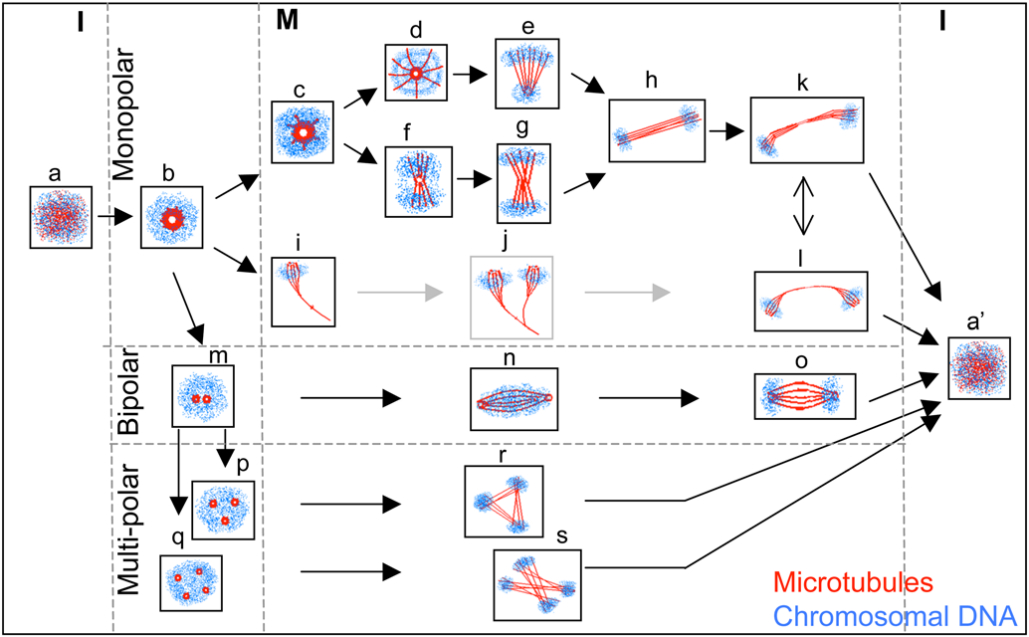
A model proposing multiple modes of chromosome segregation on diverse microtubular structures in *E. histolytica*. Based on the organization of chromosomal DNA along the different MT assemblies visualized during the nuclear cycle of axenically grown *E. histolytica* trophozoites (cell density ∼1.5–3×10^5^ cells per ml), a model for multiple modes of chromosome segregation is proposed. The schematic diagrams are adapted from the microscopic images shown in Figures 2 and 3. During interphase (I) microtubular structures are not visible (a). At the earliest stage, a single MTOC or pole is formed at the centre of the nucleus (b). Duplicated chromosomes may attach to MT fibers emanating from this pole (c). As the MT fibers elongate they carry one set of chromosomes away from the pole to which the other set is attached (d, e). In another scenario, if the two sets of chromosomes are bound to the leading end of the MTs only, then segregation may occur by lateral movement of the MT fibers (f, g). A third possibility is visualized from the lateral segregation of bundles of MTs joined at one end (i). While (j) was not actually seen in our study, we propose it as a likely intermediate between (i) and (l). Ultimately, the spindle intermediates arising from the monopolar assembly (h, k and l) may segregate into two nuclei at the completion of mitosis (M). In the case of pole duplication (m), a bipolar spindle assembly may segregate chromosomes, except that unlike typical spindles, the chromosomes are arranged along the MT fibers rather than the equatorial plate at metaphase (n, o). In polyploid nuclei, tri-polar (r) or tetra-polar spindles (s) may originate from multiple spindle poles (p and q).

This study shows that MT structures in *E. histolytica* include monopolar, bipolar and multi-polar spindles for the segregation of chromosomal DNA (Figures 2 and 3). On the basis of our findings, we propose a model (Figure 7) to explain possible modes of chromosome partitioning on these different MT structures that were identified during the nuclear cycle in axenically grown *E. histolytica* cells. It is conceivable that some of the proposed intermediate or observed MT structures are abortive. It is important to note that a large number of proteins known to regulate chromosome segregation and spindle assembly were not encoded in the amoeba genome [20]. Absence of these regulatory functions may lead to the formation of unconventional MT structures.

### Cytokinesis is uncoupled from the nuclear duplication cycle in *E. histolytica*

Cytokinetic processes in *E. histolytica* (this study) and *E. invadens* [21] are similar. Both organisms depend upon mechanical rupture of a cytoplasmic bridge that may occur independently or utilizes assistance from helper cells. Motility and the consequent mechanical force driven by actin polymerization in a polymorphic cell may have been one of the earliest modes of cell division [22]. Assistance from helper cells likely results from ‘altruistic’ behaviour of genetically related cells in a clonal population. The cytokinetic process is imprecise and irregular in *Entamoeba*, unlike the finely regulated cell division in bacteria, yeasts and higher eukaryotes. While endogenous *Entamoeba* proteins may affect the rate of cell division as deduced from increased multi-nucleation in different mutants [23]–[26], this event appears to be largely controlled by extra-cellular factors. Random selection of cell division sites coupled to a poorly controlled mechanical separation can lead to the formation of anucleate and multi-nucleated daughter cells. Similar cytokinetic events are observed in the social amoeba *D. discoideum* which shows variations in its mode of cell division. Multi-nucleated Myosin II-null *D. discoideum* cells, when allowed to adhere on substrate, undergo a cell cycle-uncoupled, inefficient cytofission [27],[28].

### The nuclear DNA content is not strictly regulated in *E. histolytica*

The nuclear cycle was clearly defined between initiation of DNA synthesis and completion of nuclear division. That a significant number of nuclei continued accumulation of DNA after doubling their DNA content emphasizes the absence of stringent regulatory mechanisms preventing re-replication that are seen in other eukaryotes. Heterogeneity of MT structures also suggests a lack of strict control of genome segregation mechanisms. It may be noted that even after serum synchronization, the maximal number of nuclei with MT structures and bi-nucleated cells were only around 20%. Treatment of *E. histolytica* cells with aphidicolin or taxol did not lead to a significant increase of nuclei in different mitotic stages (data not shown). Thus the entire nuclear cycle appears to be poorly controlled, resulting in leaky phenotypes that are apparent in polyploid nuclei. In spite of leaky regulatory mechanisms, S-phase, genome segregation and nuclear division are temporally coupled. Multiple MTOCs and multi-polar MT spindles possibly facilitate a return towards euploidy for polyploid nuclei.

Strict regulation of the cell division cycle has been considered to be the very basis of survival for eukaryotes. Indeed an unregulated cell cycle leads to growth arrest, aneuploidy and tumorigenesis in yeasts and higher eukaryotes. While prokaryotes use overlapping parallel processes of DNA synthesis, segregation and cell division, eukaryotes ensure completion of the preceding stage before initiation of the next, with tight surveillance to ensure the same. Clearly *Entamoeba* and few other organisms can bypass these surveillance mechanisms and continue their life cycles. How does *Entamoeba* preserve its genetic composition in the absence of cell cycle regulation? The answer possibly lies in its survival as a parasite, dependant on its surroundings, where plasticity and heterogeneity are favored over precision and homogeneity.

## Supporting Information

Figure S1 Effect of cell density on the growth rate of *E. histolytica* trophozoites. (A) Cell number increases continuously rather than in a step-wise fashion. Cell number was counted at different time points (0–12 h) after serum starvation and addition and plotted. Error bars indicate±S.D. (n = 3). (B) Growth rate is dependant on cell density. Log phase *E. histolytica* HM-1:IMSS cells were inoculated in TYI-S-33 medium at different cell densities- 1×10^4^, 5×10^4^ and 10×10^4^ cells/ml. Subsequent growth of these cells after 20 and 40 h is shown graphically. Error bars indicate±S.D. (n = 3).
(0.30 MB TIF)

Figure S2 Two modes of cell division in *E. histolytica*. Log phase *E. histolytica* HM-1:IMSS cells were incubated with in fresh medium at 37°C to induce cell division events. Cytokinesis was visualized under a 20× phase contrast objective of the Axiovert 200 M microscope and time-lapse images were captured at 1 sec intervals. During cell division, cytoplasmic constriction led to the extension of a cytoplasmic bridge (arrowhead) between two dividing halves. This bridge could either (A) rupture independently or (B) with the assistance of a helper or midwife cell (star). Arrows show ends of the severed cytoplasmic bridge. Bar represents 20 µm.
(3.47 MB TIF)

Video S1 Mechanical rupture of cytoplasmic extension leads to cell division in *E. histolytica*.
(3.75 MB MOV)

Video S2 Helper cell assisted cell division in *E. histolytica*.
(0.39 MB MOV)

Video S3 Aborted cytokinesis in *E. histolytica*.
(9.98 MB MOV)
