# Supplementary figures and images for: Inter-Cellular Variation in DNA Content of Entamoeba histolytica Originates from Temporal and Spatial Uncoupling of Cytokinesis from the Nuclear Cycle
Source: PLoS Negl Trop Dis. 2009 Apr 7;3(4):e409. doi: 10.1371/journal.pntd.0000409 (PMC2659751; doi:10.1371/journal.pntd.0000409)

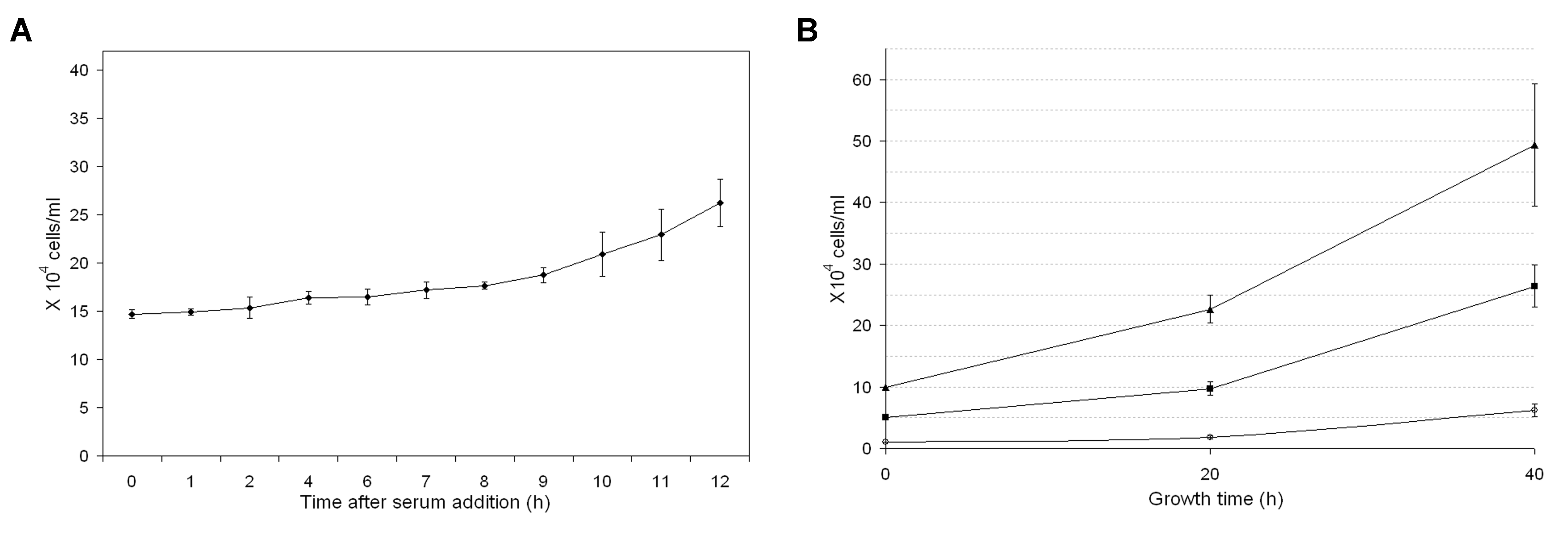

Supplement: Figure S1 — Effect of cell density on the growth rate of E. histolytica trophozoites. (A) Cell number increases continuously rather than in a step-wise fashion. Cell number was counted at different time points (0–12 h) after serum starvation and addition and plotted. Error bars indicate±S.D. (n = 3). (B) Growth rate is dependant on cell density. Log phase E. histolytica HM-1:IMSS cells were inoculated in TYI-S-33 medium at different cell densities- 1×10^4^, 5×10^4^ and 10×104 cells/ml. Subsequent growth of these cells after 20 and 40 h is shown graphically. Error bars indicate±S.D. (n = 3). (0.30 MB TIF) [file pntd.0000409.s001.tif]

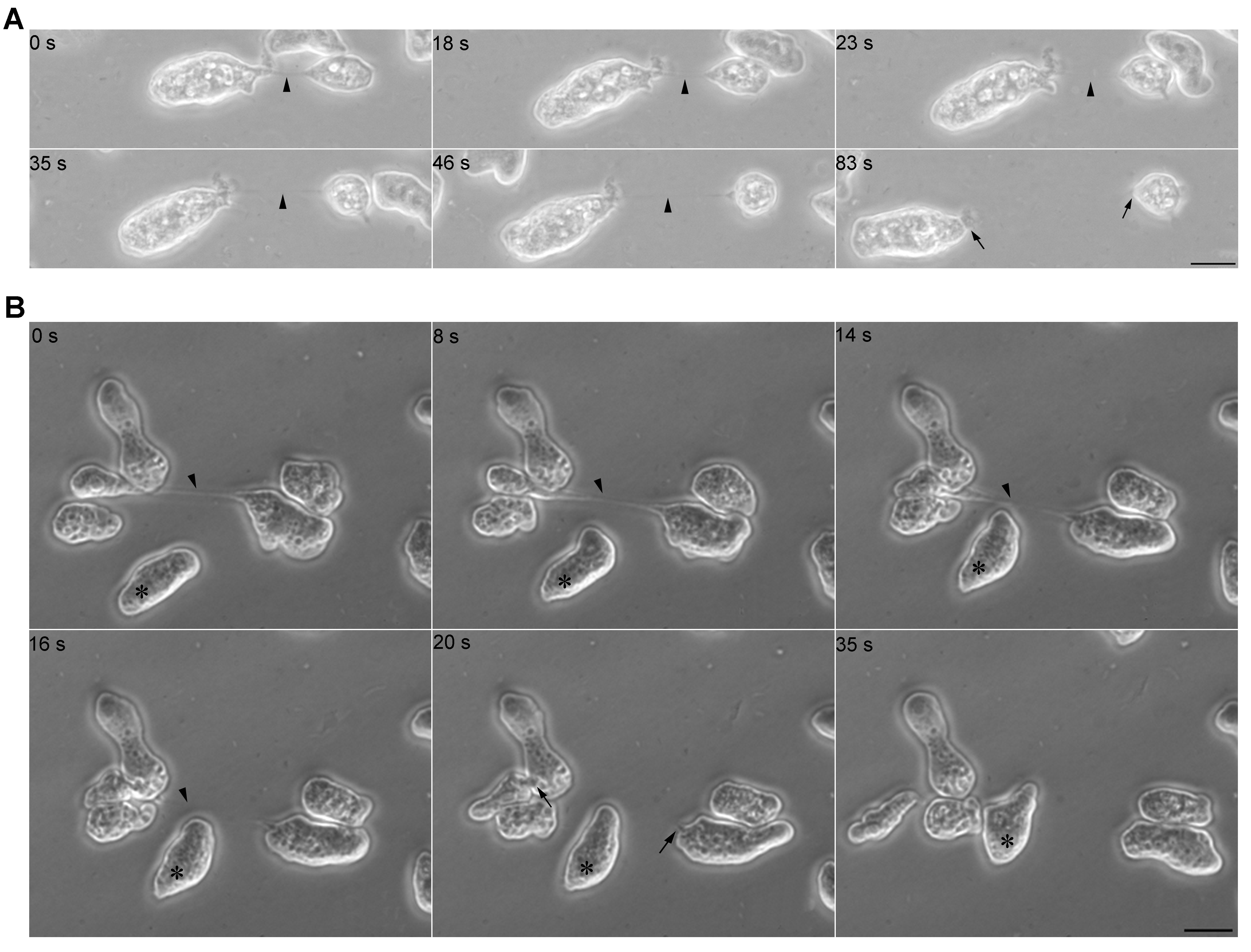

Supplement: Figure S2 — Two modes of cell division in E. histolytica. Log phase E. histolytica HM-1:IMSS cells were incubated with in fresh medium at 37°C to induce cell division events. Cytokinesis was visualized under a 20× phase contrast objective of the Axiovert 200 M microscope and time-lapse images were captured at 1 sec intervals. During cell division, cytoplasmic constriction led to the extension of a cytoplasmic bridge (arrowhead) between two dividing halves. This bridge could either (A) rupture independently or (B) with the assistance of a helper or midwife cell (star). Arrows show ends of the severed cytoplasmic bridge. Bar represents 20 µm. (3.47 MB TIF) [file pntd.0000409.s002.tif]
